# Multimodality imaging in transcatheter aortic valve implantation (TAVI): comparison between cardiovascular magnetic resonance, cardiac computed tomography and echocardiography

**DOI:** 10.1186/1532-429X-13-S1-O51

**Published:** 2011-02-02

**Authors:** Jabbour Andrew, Francois Okoroafor, Bradley Park, Isabelle Roussin, Francisco Alpendurada, Tevfik Ismail, Maria N Paes, Simon Davies, Michael J Mullen, Nicola Delahunty, Sanjay Prasad, Michael Rubens, Neil Moat, Raad Mohiaddin

**Affiliations:** 1 Royal Brompton Hospital and Imperial College, London, UK; 2 Royal Brompton Hospital, London, UK; 3 Imperial College, London, UK

## Background

Patients considered for TAVI often undergo several cardiac imaging investigations during assessment. Although each imaging modality has its particular advantages, not all imaging modalities are universally available. This study sought to determine the agreement and variability of cardiovascular magnetic resonance (CMR), electrocardiograph-gated cardiac computed tomography (cardiac CT) and transthoracic echocardiography in the assessment of aortic root size and morphology.

## Methods

Patients undergoing TAVI assessment with CMR, cardiac CT and echocardiography, were recruited to the study. Agreement and variability between each imaging modality in the measurement of aortic annulus, sinus of valsalva, sinotubular junction and ascending aorta dimensions was assessed by Bland-Altman analysis. Intraobserver and interobserver variability was also assessed and compared.

## Results

Of 201 patients undergoing TAVI assessment with both CMR and Echocardiography, 133 also underwent an ECG-gated Cardiac CT scan. Close agreement was observed between CMR and Cardiac CT in the assessment of aortic annulus dimensions (Bias -0.4 mm, SD of Bias 2.7mm, 95% Limits of agreement -5.7mm to 5.0mm), sinus of valsalva dimensions (Bias -0.6 mm, SD of Bias 2.5mm, 95% Limits of agreement -4.3mm to 5.5mm), sinotubular junction dimensions (Bias -0.7 mm, SD of Bias 2.4mm, 95% Limits of agreement -5.3mm to 3.9mm), and ascending aorta dimensions at the level of the right pulmonary artery (Bias -0.1 mm, SD of Bias 2.6mm, 95% Limits of agreement -5.3mm to 5.1mm).

Agreement between echocardiography-derived measures and either CMR or Cardiac CT was less tight. CMR to echocardiography agreement in aortic annulus dimensions (Bias -4.0 mm, SD of Bias 6.5mm, 95% Limits of agreement -16.7mm to 8.8mm), sinus of valsalva dimensions (Bias -0.7 mm, SD of Bias 4.5mm, 95% Limits of agreement -9.6mm to 8.1mm), sinotubular junction dimensions (Bias -2.1 mm, SD of Bias 4.7mm, 95% Limits of agreement -11.2mm to 7.1mm), and ascending aorta dimensions at the level of the right pulmonary artery (Bias -0.0 mm, SD of Bias 4.0mm, 95% Limits of agreement -7.8mm to 7.8mm). Intraobserver and interobserver variability was lowest in CMR-derived measures followed by Cardiac CT then transthoracic echocardiography.

## Conclusions

In patients undergoing assessment for TAVI, close agreement exists between CMR and ECG-gated Cardiac CT in the assessment of aortic root dimensions. Low intraobserver and interobserver variability was seen in both modalities, although best with CMR. Lower agreement and higher variability was observed between echocardiography and the other two imaging modalities.

